# Vacuum-assisted excision: a safe minimally invasive option for benign phyllodes tumor diagnosis and treatment—a systematic review and meta-analysis

**DOI:** 10.3389/fonc.2024.1394116

**Published:** 2024-05-14

**Authors:** Maria Luísa Braga Vieira Gil, Bertha Andrade Coelho, Henrique Lima Couto, Henrique Moraes Salvador Silva, Eduardo Carvalho Pessoa, Nisha Sharma, Ritse Mann, Stuart A. McIntosh, Paulo Henrique Costa Diniz, Farley Soares Cantidio, Gabriel Oliveira Bernardes Gil, Anna Dias Salvador, Waldeir José de Almeida Júnior, José Tadeu Campos Avelar, Cláudia Lourdes Soares Laranjeira, Agnaldo Lopes Silva Filho

**Affiliations:** ^1^ Mastology Department, Rede Mater Dei de Saúde, Belo Horizonte, Minas Gerais, Brazil; ^2^ Breast Imaging Department, Brazilian Society of Mastology, Rio de Janeiro, Rio de Janeiro, Brazil; ^3^ Department of Obtetrics and Gynecology, UNIFIMOC University Center, Montes Claros, Minas Gerais, Brazil; ^4^ Breast Imaging Department, Brazilian Federation of Associations of Gynecologists and Obstetricians, Rio de Janeiro, Rio de Janeiro, Brazil; ^5^ Redimama-Redimasto, Belo Horizonte, Minas Gerais, Brazil; ^6^ Department of Obstetrics and Gynecology, Botucatu Medical School, Sao Paulo State University (UNESP), Botucatu, Sao Paulo, Brazil; ^7^ Breast Screening Unit, Seacroft Hospital, Leeds Teaching Hospital National Health Service (NHS) Trust, Leeds, United Kingdom; ^8^ Department of Medical Imaging, Radboud University Medical Center, Nijmegen, Netherlands; ^9^ Department of Radiology, The Netherlands Cancer Institute, Amsterdam, Netherlands; ^10^ Patrick G Johnston Centre for Cancer Research, Queen’s University Belfast, Belfast, United Kingdom; ^11^ Oncology Department, Hospital Mater Dei, Belo Horizonte, Minas Gerais, Brazil; ^12^ Internal Medicine Department, Federal University of Minas Gerais, Belo Horizonte, Minas Gerais, Brazil; ^13^ Radiotherapy Department, Hospital Mater Dei, Belo Horizonte, Minas Gerais, Brazil; ^14^ Department of Obstetrics and Gynecology, Faculty of Medical Sciences of Minas Gerais, Belo Horizonte, Minas Gerais, Brazil; ^15^ Obstetrics and Gynecology Department, Rede Mater Dei de Saúde, Belo Horizonte, Minas Gerais, Brazil; ^16^ Department of Obstetrics and Gynecology, Federal University of Minas Gerais, Belo Horizonte, Minas Gerais, Brazil

**Keywords:** phyllodes tumor, vacuum-assisted excision, vacuum-assisted biopsy, local recurrence, meta-analysis, review

## Abstract

**Synopsis:**

This is a systematic review and meta-analysis comparing surgical excision with percutaneous ultrasound-guided vacuum-assisted excision (US-VAE) for the treatment of benign phyllodes tumor (PT) using local recurrence (LR) as the endpoint.

**Objective:**

To determine the frequency of local recurrence (LR) of benign phyllodes tumor (PT) after ultrasound-guided vacuum-assisted excision (US-VAE) compared to the frequency of LR after surgical excision.

**Method:**

A systematic review and meta-analysis [following the Preferred Reporting Items for Systematic Reviews and Meta-Analyses (PRISMA) standard] was conducted by comparing LR in women older than 18 years treated for benign PT by US-VAE compared with local surgical excision with at least 12 months of follow-up. Studies were retrieved from PubMed, Scopus, Web of Science, and Embase. The pooled effect measure used was the odds ratio (OR) of recurrence.

**Results:**

Five comparative prospective or retrospective observational studies published between January 1, 1992, and January 10, 2022, comparing surgical excision with percutaneous US-VAE for LR of benign PT met the selection criteria. Four were retrospective observational cohorts, and one was a prospective observational cohort. A total of 778 women were followed up. Of them, 439 (56.4%) underwent local surgical excision, and 339 (43.6%) patients had US-VAE. The median age of patients in the five studies ranged from 33.7 to 39 years; the median size ranged from 1.5 cm to 3.0 cm, and the median follow-up ranged from 12 months to 46.6 months. The needle gauge ranged from 7G to 11G. LR rates were not statically significant between US-VAE and surgical excision (41 of 339 *versus* 34 of 439; OR 1.3; p = 0.29).

**Conclusion:**

This meta-analysis suggests that using US-VAE for the removal of benign PT does not increase local regional recurrence and is a safe minimally invasive therapeutic option.

**Systematic review registration:**

https://www.crd.york.ac.uk/prospero/, identifier CRD42022309782.

## Introduction

Phyllodes tumor (PT) of the breast is a rare fibroepithelial neoplasm, accounting for 0.3% to 1% of all breast tumors and 2% to 3% of all fibroepithelial breast lesions (1, 2). PTs are classified as benign (Grade 1), borderline (Grade 2), or malignant (Grade 3) by the World Health Organization (WHO), according to histological features such as mitotic activity, the degree of stromal cellular atypia, infiltrative tumor margins, and stromal overgrowth (3). PT may contain foci with benign, borderline, and malignant features intermingled within the same neoplasm, making careful gross examination and histologic sampling particularly important. Therefore, given PT’s histologic heterogeneity and its propensity to rapidly grow, excision is required to accurately classify, grade, and treat.

The majority of PTs behave in a benign fashion, with the risk of local recurrence (LR) ranging from 17% in benign PT to 27% in malignant PT (4). Several studies questioned the necessity for large margins in benign PT, suggesting that surgical margins less than 1 cm are sufficient (5–8). Based on retrospective data, the National Comprehensive Cancer Network (NCCN) recommends wide local excision (WLE) with a 1-cm margin or more for borderline/malignant PT, but excisional biopsy for benign PT is acceptable (9).

Recently, many studies have shown that complete removal of benign PT using percutaneous excision is feasible (10–14). With this procedure, PTs are removed sample by sample in a piecemeal fashion using 7- to 11-G vacuum-assisted needles under ultrasound guidance (US-VAE). It was postulated that a “wait and watch” approach after vacuum excision (without evident clear margins) could be a safe alternative to local surgical excision with low LR (14, 15). Indeed, complete percutaneous removal would safely exclude a malignant diagnosis and may result in acceptable local control for benign PT.

The present study evaluates whether the LR after US-VAE is different from that observed after surgical excision to determine if US-VAE is a safe minimally invasive option for benign PT treatment.

## Methods

### Study design and selection criteria

A systematic review and meta-analysis, following the Preferred Reporting Items for Systematic Reviews and Meta-Analyses (PRISMA) standard, was conducted (16). LR was compared in women older than 18 years treated for benign PT by US-VAE or local surgical excision with at least 12 months of follow-up.

### Search strategy

The following keywords, terms, and their combinations were used to formulate the search strategy: phyllodes tumor, phylloid tumor, vacuum-assisted excision, vacuum biopsy, and vacuum-assisted biopsy. A broad search in PubMed, Scopus, Web of Science, and Embase databases using these keywords combined with Boolean AND or OR, for articles published between January 1, 1992, and January 10, 2022, was conducted on January 11, 2022. The research was limited to human studies. There was no language restriction.

### Selection of studies

Papers that described randomized clinical trials and comparative observational prospective and retrospective studies clearly presenting the rate of local recurrence for both groups were selected.

The studies identified in the initial search were imported into the Rayyan app for sorting and selecting articles (17). Two researchers independently and blindly selected relevant articles using Rayyan. Initially, the studies were grouped, duplicate articles were removed, and conference summaries, editorials, comments, letters, and case reports were excluded. Discordance in the selected articles was resolved by consensus, and when this was not attained, a third researcher acted as an arbiter. The complete papers of potentially relevant articles were retrieved and evaluated for eligibility. Duplicated papers were removed.

Only articles where complete US-VAE resection was defined as the complete removal of the lesion, verified by clinical examination (absence of palpable nodule) and ultrasound (absence of any lesion visualized in real-time examination), were selected. A clinically palpable lesion in the breast or its observation on ultrasound in a previous tumor resection bed was considered a local recurrence. Lesions more than 2 cm away from the original tumor were not considered recurrences. Therefore, only studies that provided sufficiently detailed information were included. The two researchers independently decided on the inclusion or exclusion of studies based on the predefined inclusion and exclusion criteria; again, a third researcher acted as an arbiter. The reasons for the exclusion of any article were documented.

### Data extraction

Data from eligible studies were extracted independently by the researchers using a standard form for data extraction developed for this review. The form contains the characteristics of the study (design), the participants (sample size and age), interventions, outcomes assessed, and the duration of follow-up (see **Supplementary Material**).

### Assessment of methodological quality

The risk of bias in the studies was assessed through the specific forms from the Joanna Briggs cohort study Institute—JBI Critical Appraisal checklist, 2020 (18). The analysis of the publication bias was carried out through the inspection of data asymmetry, according to the dispersion and size of the effect in the funnel plot.

### Statistical analysis

According to the binary variable analyzed, the LR of benign PT and odds ratio (OR) were calculated as an effect estimation measure. For this purpose, the Mantel–Haenszel test was used. A fixed or random effect was applied according to the heterogeneity index found. All analyses were performed on RevMan Web. For the evaluation of heterogeneity, Higgins’ heterogeneity classification was used. I^2^ < 40% represents inexpressive heterogeneity, whereas values above 75% indicate high heterogeneity. Following the recommendations, the statistical analysis was adjusted according to the level of heterogeneity, applying a fixed-effects model for inexpressive heterogeneity (low) and a random-effects model for high heterogeneity (18).

## Results

**Figure 1:**
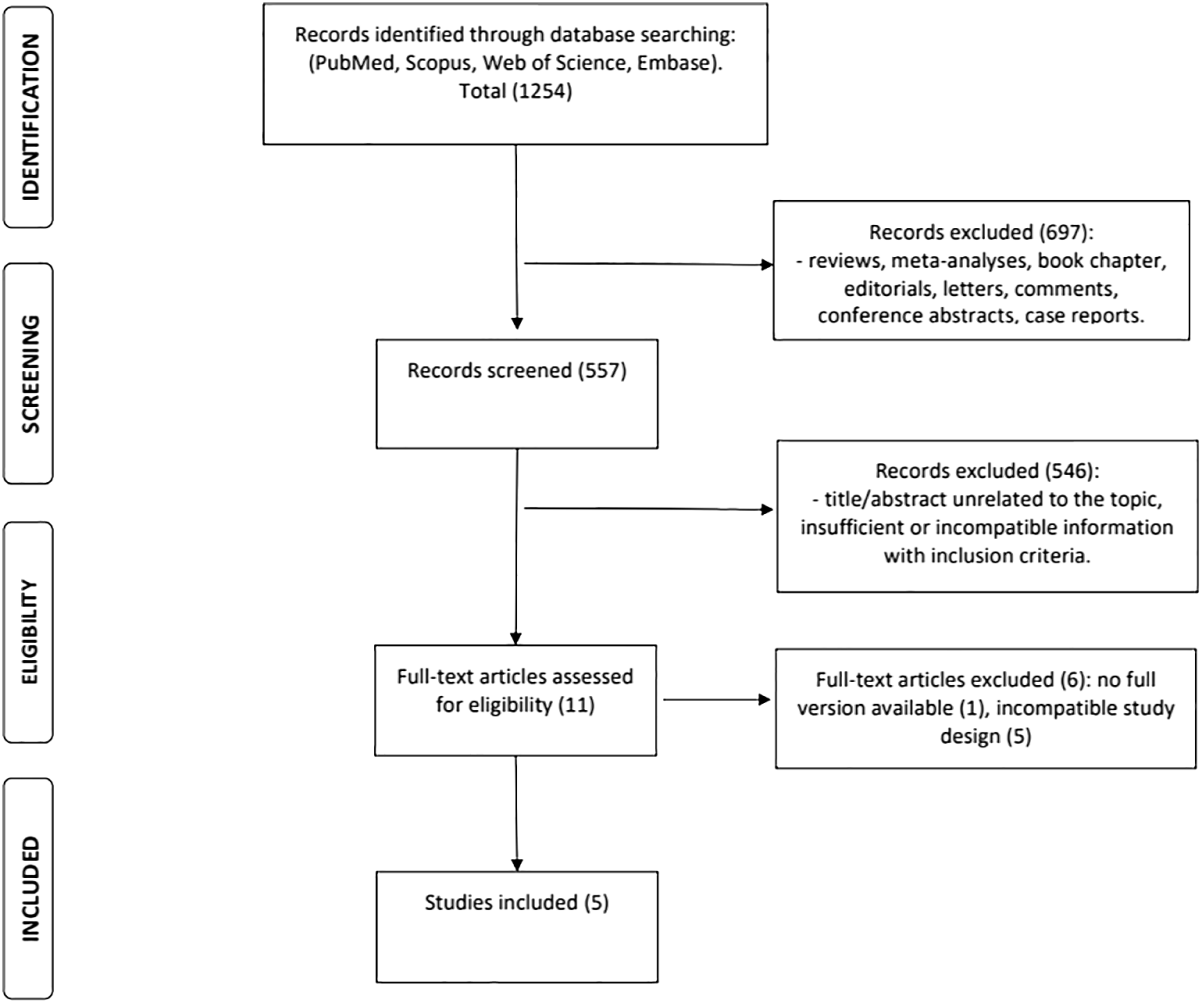
Flowchart of the search results and article selection.

A total of 1,254 references published between January 1, 1992, and January 10, 2022, were identified. Reviews, conference abstracts, editorials, commentaries, letters, and case reports were also excluded (n = 697). Titles and abstracts were examined to remove articles that were not related to the topic (n = 546). The full texts of potentially relevant articles (n = 11) were retrieved, and the eligibility criteria were evaluated. Six articles were excluded: five did not present the comparator group (surgery) and/or presented incomplete data on recurrence rates following surgery, and one was published in a Chinese periodical that was not available. Finally, all five articles selected were observational cohorts: four retrospective and one prospective (**Figure 1**). Four papers were from Asia: three from China and one from Korea. One paper was from the USA (**Table 1**).

**Table 1:** Results from systematic review.

Author	Ji et al.	Chao et al.	Ouyang et al.	Kim et al.	Vargas et al.
**Year**	2022	2020	2016	2016	2006
**Location**	China	China	China	South Korea	United States
**Study design**	Retrospective observational cohort	Retrospective observational cohort	Retrospective observational cohort	Retrospective observational cohort	Prospective observational cohort
**Period**	2006–2020	2005–2014	2005–2013	2002–2012	2001–2003
Age (years)*	39 ± 11.7	38	Surgery: 38 US-VAE: 36.5	38.5 (16–71) Surgery: 39 (16–71) US-VAE: 33 (19–49)	33.74 ± 11.5 (15–68)
**Patients (n)**	171	224	225	146	12
**US-VAE group (n)**	87	119	108	20	5
**Surgery group (n)**	84	105	117	126	7
**US-VAE group’s recurrence**	15	14	12	0	0
**Surgery group’s recurrence**	10	13	8	3	0
**Follow-up period (months)**	48.6 (3–107)	37 (15 a 208)	35.5 (12-120)	32.3 (6.7–142.5)	12
**Interval to recurrence (median/months)**	36	28	21.8/22.5	17.4	-
**Tumor size US-VAE group (median/cm)**	2 > T < 5 Median range	2 > T < 5 Median range	1.7	1.6 (0.3–3.9)	1.57 cm (0.3–4.0) Overall cohort
**Tumor size Surgery group (median/cm)**	-	-	3	2.5 (0.7–2.3)	-
**VAE**	EnCor SenoRx^®^	Did not specify	Mammotome^®^	Mammotome^®^	Mammotome^®^
**Gauge**	7	Did not specify	8	8 or 11	8 or 11
**Surgery type**	Lumpectomy (surgical margin <1 cm), wide excision or mastectomy (margin > 1 cm)	Lumpectomy (surgical margin <1 cm)	Did not specify	Did not specify	Did not specify

US-VAE, ultrasound-guided vacuum-assisted excision; VAE, vacuum-assisted excision.

*Mean ± SD; median or range.

Therefore, the meta-analysis comprised the pooling of five studies, compiling a total of 778 participants with benign PT treated by excisional surgery or US-VAE between 2001 and 2020. Of these patients, 439 (56.4%) had local surgical excision, and 339 (43.6%) patients had US-VAE. The median age of patients in the five studies ranged from 33.7 to 39 years; the median T size ranged from 1.5 cm to 3.0 cm, and the median follow-up ranged from 12 months to 46 months (**Table 1**).

Three studies (Chao et al., 2020, Vargas et al., 2006, and Ji et al., 2022) (11, 12, 19) did not present the Breast Imaging-Reporting and Data System (BI-RADS) classification of the lesions. Ouyang et al. (2016) (14) reported that most patients had BI-RADS 4 lesions in the surgery group (58.1%), and Kim et al. (2016) (20) reported 57.1%. In the US-VAE group, most lesions were classified as BI-RADS 2–3 (58.3%) by Ouyang et al. (2016) (14) and 50% in the study by Kim et al. (2016) (20). Only one study presented the BI-RADS classification of the relapse (Kim et al., 2016) (20). All recurrences of this study occurred in the surgery group and were classified as BI-RADS 4.

**Figure 2:**
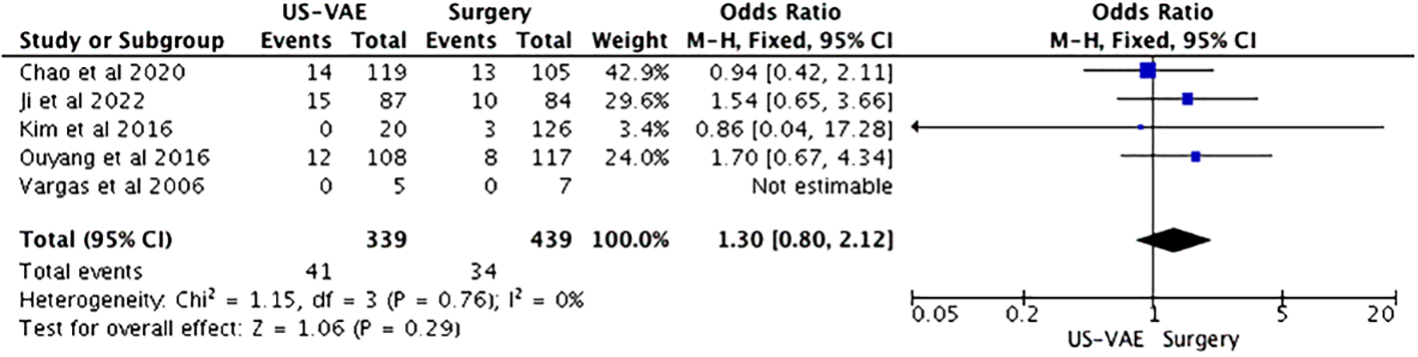
Forest plot of the included studies analyzing the chance of benign PT recurrence. Total of patients = 778. PT, phyllodes tumor.

In the US-VAE group (n = 339), local recurrence was observed in 41 (12.1%) cases, and in the surgery group (n = 439), 34 (7.7%) cases. There was no significant difference in benign PT local recurrence regardless of the procedure performed (OR = 1.30; p = 0.29) (**Figure 2**).

### Quality analysis and risk of bias

**Figure 3:**
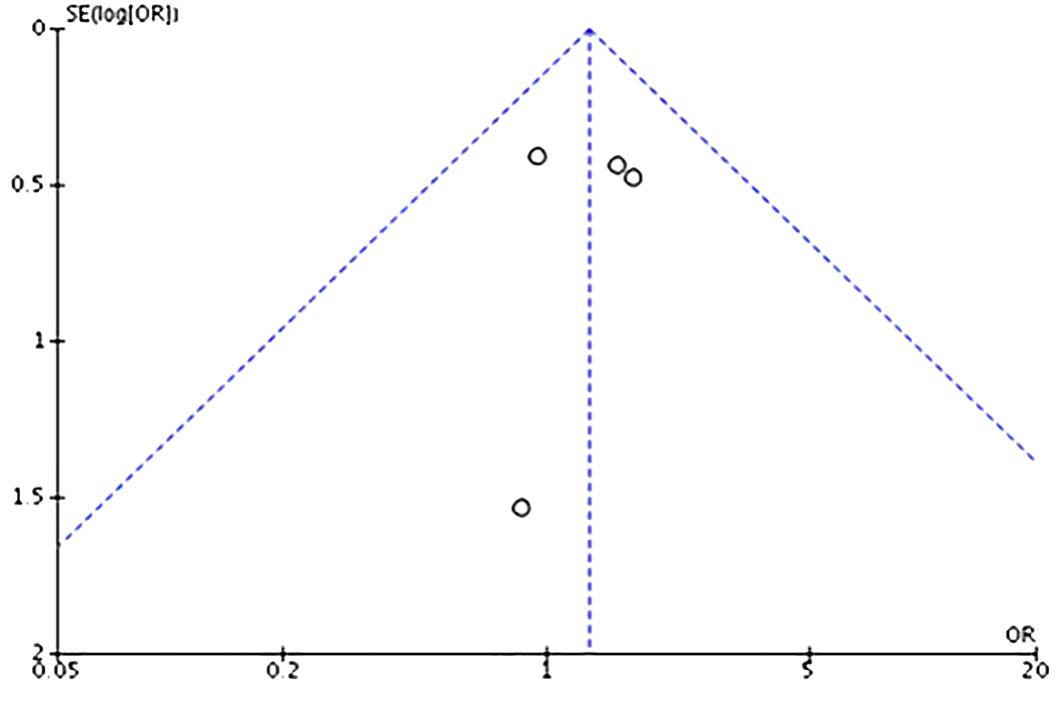
Funnel plot analysis for symmetry (no publication bias) of the included studies.

There was no significant publication bias, according to individual analysis of the studies (**Table 2**) and by visual inspection of the funnel plot (**Figure 3**), which shows no asymmetry of the studies in their positions in the funnel. One of the studies was not plotted because no case of recurrence occurred in both conditions (US-VAE and surgery).

**Table 2:** Risk of individual bias in the studies.

Author/year	Vargas et al., 2006 (19)	Kim et al., 2016 (20)	Ouyang et al., 2016 (14)	Chao et al., 2020 (11)	Ji et al., 2022 (12)
**1. Similar groups and recruited from the same population?**	Yes	Yes	Yes	Yes	Yes
**2. Similar inclusion criteria among the groups?**	Yes	Yes	Yes	Yes	Yes
**3. Quickly and reliably evaluated exposure?**	Yes	Yes	Yes	Yes	Yes
**4. Confounding factors identified?**	Yes	Yes	Yes	Yes	Yes
**5. Strategies to deal with confounding factors?**	No	Yes	Yes	Yes	Yes
**6. Participants free of the outcome at the beginning of the study (or at the time of exposure)?**	Yes	Yes	Yes	Yes	Yes
**7. Outcomes evaluated in a valid and reliable way?**	Yes	Yes	Yes	Yes	Yes
**8. Reported follow-up time long enough for the outcomes to occur?**	Yes	Yes	Yes	Yes	Yes
**9. Time tracking complete? Reasons for loss of described?**	Yes	Yes	Yes	Yes	Yes
**10. Strategies for addressing loss to follow-up?**	Yes	Yes	Yes	Yes	Yes
**11. The appropriate statistical analysis?**	Obscure	Yes	Yes	Yes	Yes
**Overall evaluation**	Included	Included	Included	Included	Included

## Discussion

There are no randomized data, nor are there ever likely to be, comparing US-VAE, margins < 1 cm, and margins > 1 cm for surgical treatment of benign PT. Our meta-analysis supports US-VAE as an alternative approach to surgery for benign PT, as there is no significant difference in LR after resection by either surgical excision or US-VAE. The current diagnosis and management of PT are based on observational data (1–9). Pathologists find it challenging to exclude malignant phyllodes on core needle biopsy (CNB)/vacuum-assisted biopsy (VAB) sampling and tend to classify them as fibroepithelial lesions. Current practice consists of excisional surgery with clear margins, but not 2 cm wide, as most of these cases are fibroadenoma or benign PT (9). There are emerging data supporting the so-called “wait and watch” approach that consists of follow-up and observation after surgical excision with positive margins or a US-VAE (10–15, 21), and our meta-analysis corroborates this.

The usual clinical scenario is a mass, palpable or not, seen on ultrasound that is submitted to CNB or VAB, yielding an inconclusive result, usually a cellular fibroepithelial lesion (22, 23). These lesions are classified as lesions of uncertain malignant potential (B3 lesions) and were historically always excised. A large fraction (up to 69%) of these lesions turn out to be cellular fibroadenomas (22, 23). Only a very small proportion (up to 2%) of lesions diagnosed as cellular fibroepithelial lesions are malignant PT in the excision specimen (22–24), although the literature shows a wide variation (16% to 76%) of the “upgrade rate” to benign PT on excision (23–26). Another common clinical scenario is a mass, palpable or not, seen on ultrasound that had been submitted to CNB/VAB and was diagnosed as fibroadenoma but shows significant growth over time, raising the possibility of a misdiagnosed PT.

Some or even most of the benign PTs occasionally found in breast cancer screening are round, circumscribed, non-palpable masses categorized as American College of Radiology (ACR) BI-RADS 3 and submitted to follow-up without any percutaneous diagnostic procedure. However, they grow over time, as a rule, and then will be indicated for investigation, and the previously described clinical–pathological situations arise.

**Figure 4:**
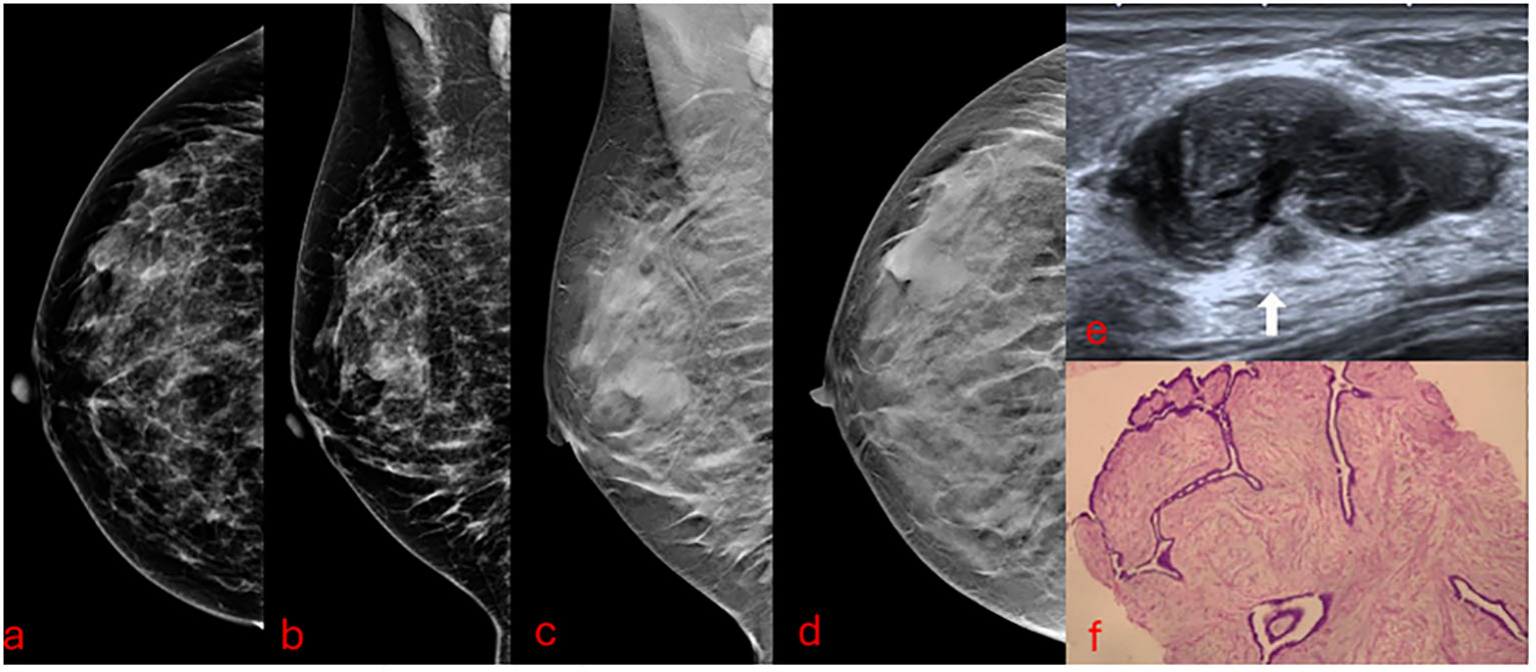
A 39-year-old patient presenting a palpable developing mass of 29 mm in the superior outer quadrant of the right breast with previous core needle biopsy of benign PT submitted to US-VAE has been followed up for 3 years without recurrence. **(A, B)** Mammograms. **(C, D)** Digital breast tomosynthesis. **(E)** Ultrasound. The arrow points to the mass that proved to be a benign PT. **(F)** VAE core sample slide in low-power microscopic field (×40). PT, phyllodes tumor; US-VAE, ultrasound-guided vacuum-assisted excision; VAE, vacuum-assisted excision.

**Figure 5:**
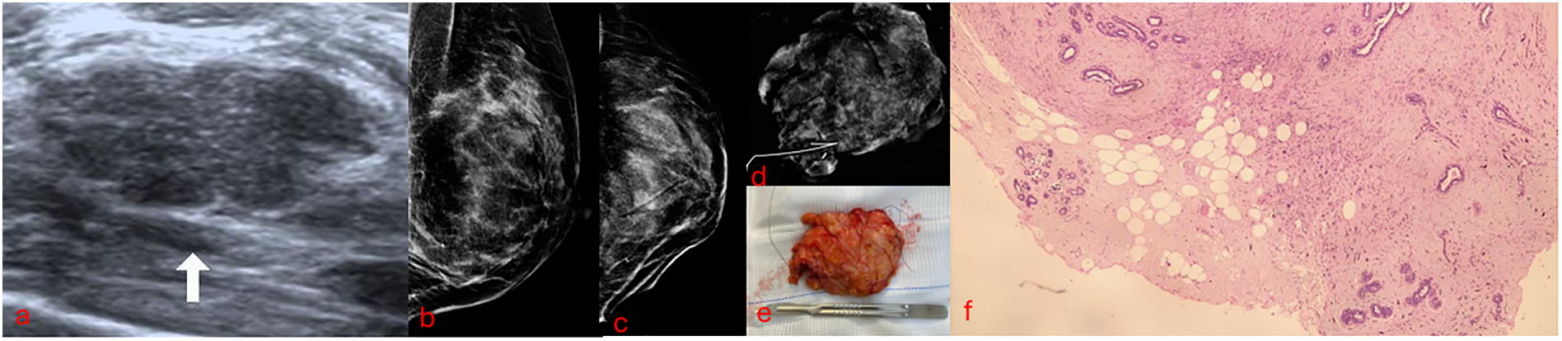
A 45-year-old patient with a developing non-palpable left breast mass of 23 mm initially categorized as ACR BI-RADS 3 diagnosed as fibroadenoma on core needle biopsy. US-VAE diagnosed a borderline PT. Surgery revealed a 3-mm residual borderline PT. **(A)** Ultrasound. The arrow points to the mass that proved to be a borderline PT. **(B, C)** Mammograms soon after US-VAE confirmed a successful procedure by no residual lesion and marker well positioned. **(D)** Surgical excision radiography; the marker proves the precise excision of previous US-VAE site. **(E)** Lumpectomy surgical specimen. **(F)** Low-power microscopic field of borderline PT showing focal infiltrative borders. ACR BI-RADS, American College of Radiology Breast Imaging-Reporting and Data System; US-VAE, ultrasound-guided vacuum-assisted excision; PT, phyllodes tumor.

**Figure 6:**
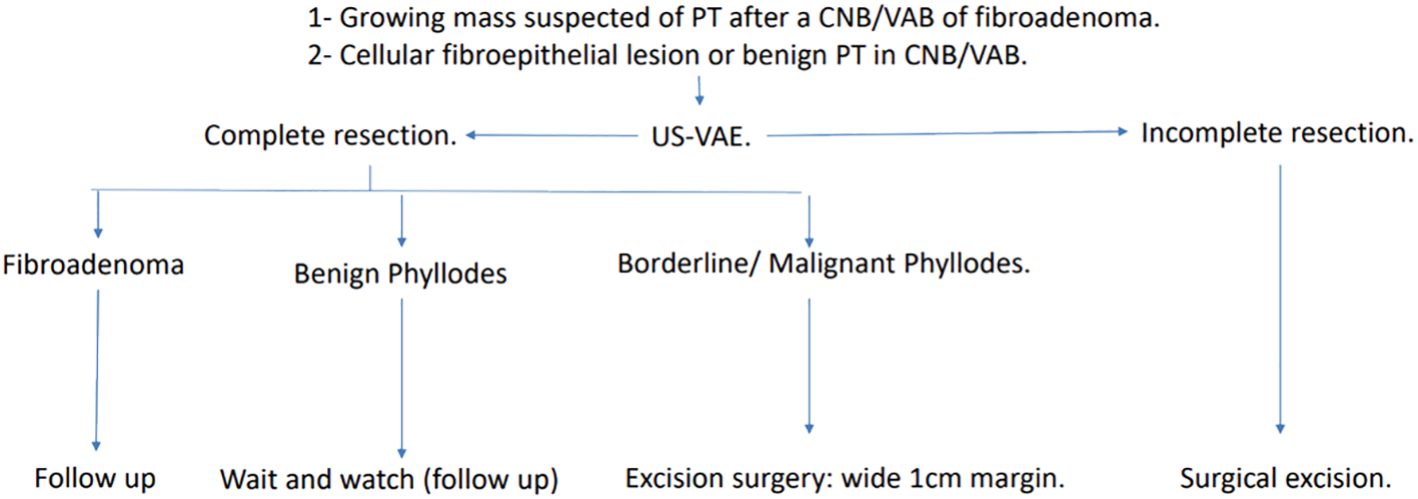
Pathway proposed for managing lesions suspected of benign PT or diagnosed as cellular fibroepithelial lesions or benign PT in CNB/VAB. Complete US-VAE resection: absence of residual imaging findings and palpable mass after a successful procedure. Incomplete US-VAE resection: residual imaging findings or palpable mass or even an unsuccessful procedure. PT, phyllodes tumor; CNB/VAB, core needle biopsy/vacuum-assisted biopsy; US-VAE, ultrasound-guided vacuum-assisted excision.

In current practice, to exclude malignancy and treat benign PT, the next step in the scenarios above is surgical excision. Most of these excisions result in the diagnosis of benign breast tissue or cellular fibroadenomas (22–26) and could be avoided if a conclusive percutaneous diagnosis with US-VAE was achieved. This analysis shows that even when after US-VAE a benign PT is diagnosed, many surgical excisions could still be avoided using a “wait and watch” approach (27) (**Figure 4**), and borderline/malignant PT would not be missed (**Figure 5**). Based on this, we propose a new pathway for lesions submitted to CNB/VAB with a diagnosis of cellular fibroepithelial lesion or benign PT and for lesions with a diagnostic CNB/VAB of fibroadenoma but showing significant growth over time, raising the possibility of PT (**Figure 6**).

Surgical management of PT is a controversial subject in the literature (2). Wide local excision with minimal 10-mm safe margins is currently being suggested for all subtypes of PT (2). The NCCN guideline (2) recommends wide local excision with the intention of obtaining margins of 1 cm or more for each PT grade. However, their supporting evidence came from a retrospective study that was limited by a small sample size at a single institution (28), and they have recommended that there is no need for re-excision if free margins are achieved for benign PT. Benign PT should be managed differently than borderline/malignant PT (21). There is evidence suggesting that margins < 10 mm or even positive margins do not affect LR for benign PT (21, 29–31). Indeed, a recently published meta-analysis demonstrated that a positive surgical margin was significantly associated with a higher LR risk for malignant PTs, but only a tendency for an increase in the LR risk was observed for benign and borderline PTs (21). In the case of positive margins, there is still the possibility of no re-excision and follow-up: the so-called “wait and watch” strategy (9, 21, 27, 32–34).

There is no relation between margins and distant metastasis for PT, irrespective of grade (benign, borderline, and malignant). Therefore, it is undeniable that breast conservative surgery is the surgical treatment of choice for all subtypes of PT provided that the excised breast volume due to the tumor size does not induce breast deformity (30, 35). Especially for benign PT, a more conservative approach would be appropriate. Our meta-analysis supports the assumption that the proposed pathway is safe.

The upper tumor size limit to indicate a US-VAE for benign PT, and therefore for masses with a CNB/VAB diagnostic of cellular fibroepithelial lesions or benign PT, is controversial and could not be precisely addressed by this meta-analysis. In one study (Chao et al., 2020) (11), tumor size was presented in ranges (2 cm, >2 cm to 5 cm, and >5 cm), and 59% of cases were classified as measuring >2 cm to 5 cm. The mean tumor size at recurrence was not presented in this study. Ji et al. (2022) (12) also presented the tumor size by ranges, with most cases (63.7%) classified as measuring >2 cm to 5 cm. The median tumor size was 2.5 cm between the groups of benign phyllodes tumors treated by US-VAE or surgery. Vargas et al. (2006) (19) presented only the overall mean tumor size (1.57 ± 0.61 cm; 0.3–4.0), which did not differ between the groups (19) (**Table 1**). In line with these results, if US-VAE complete resection of the visible lesion is achieved, based on the findings of this meta-analysis, further surgical resection seems unnecessary. These findings reinforce recommendations of The Third International Consensus Conference on lesions of uncertain malignant potential in the breast (B3 lesions) that have recognized follow-up as an option for benign PT submitted to US-VAE (36).

There are some limitations to our meta-analysis. Only two studies reported median tumor size in the two groups: 1.7 cm for US-VAE and 3.0 cm for surgery (Ouyang et al., 2016) (14) and 1.6 cm for US-VAE and 3.0 cm for surgery (Kim et al., 2016) (20). Although the median tumor size was smaller in the US-VAE group than in the surgery group, there were no associations between tumor size and local recurrence. Still, the studies are all non-randomized cohorts, and therefore, there is the potential for selection bias in the cases having US-VAE *versus* those having surgery. The selection criteria to indicate US-VAE or surgery in each of the five institutions of the studies were not completely outlined. US-VAE is a relatively new and not well-established approach. It is possible that some kind of selection bias favored the US-VAE arm in the studies.

Regarding the number of patients and events, this is the largest series (the only meta-analysis) that has addressed US-VAE × surgery for benign PT LR. Although not statistically significant, the LR was higher in the US-VAE group. There was a trend favoring surgery in terms of LR, but there is no guarantee that this is going to be confirmed with a larger cohort. Prospective registration of long-term outcomes for centers that will adopt US-VAE as an alternative to surgery for cellular fibroepithelial lesions and benign PT is recommended.

In addition, surgical procedures are not described in detail. Two studies (Chao et al., 2020 and Ji et al., 2022) (11, 12) described tumorectomy as the excision of the nodule with a surgical margin < 1 cm, wide local excision with a margin > 1 cm, and conservative surgery and mastectomy with a margin ≥ 1 cm. In three studies (Kim et al., 2016; Ouyang et al., 2016; Vargas et al., 2006) (14, 19, 20), the type of surgery performed was not specified. With the available data, it is not possible to analyze the relation of margin width and recurrence between US-VAE and surgery.

There is uncertainty on the pathological diagnostic accuracy of complete US-VAE mass resection for cellular fibroepithelial lesions and PT. PT is classified into three grades according to the WHO: benign, borderline, and malignant. The histological features that are used to classify PT are stromal cellularity, stromal atypia, mitosis, stromal overgrowth, and tumor margin (3). These are also histological features related to LR (21). Three of these could have impaired evaluation. Tumor margin (well-defined, well-defined or focal infiltrative, or infiltrative) could be underestimated, nevertheless not probable to occur. Histological core samples of complete US-VAE provide an evaluation of the borders of the PT, which consists of PT and normal breast tissue, so the microscopic invasion is still possible to evaluate (**Figures 4**, **5**). Stromal overgrowth, defined by the absence of epithelial elements in one low-power microscopic field containing only stroma (3), could be underestimated or overestimated because vacuum core samples are smaller than a low-power field. Stromal cellularity also could be underestimated or overestimated because of the fragmentation. There would be some challenges to distinguishing between borderline and malignant PT, although benign PT is the low spectrum, and there would be no challenge to its diagnostic (**Figure 4**).

Furthermore, needle sizes between studies were different. One study used 7-gauge needles (G), two used 11 G or 8 G, and one used 8 G, and one study did not specify. In Vargas et al. (2006) (19), the 8-G needle was used in 169 patients, and the 11-G needle was used in 41 patients. One study (Ouyang et al., 2016) (14) used only 8-G needles. It was not possible to determine the relation between the needle gauge and LR. Probably, the gauge of the needle used does not matter if complete excision is achieved.

Finally, the recurrence intervals between the US-VAE and surgery groups described in **Table 1** are in general relatively short and not well described. The study by Chao et al. (2020) (11) presents only the mean overall recurrence interval. It does not differentiate it by groups. Ji et al. (2022) (12) presented only the mean overall recurrence interval (36 months) and did not differentiate it by groups. It is important to realize that in a recent meta-analysis evaluating LR of PT, the median time to recurrence was longer than 24 months in nine studies and shorter than 24 months in eight studies; one of the studies presented a median local recurrence time of 6 months (21). In our meta-analysis, the median follow-up time from the studies ranged from 12 to 48.6 months, and the mean time to recurrence ranged from 17.4 to 36 months. In one of the studies, the follow-up time was just 12 months but showed no recurrence at all in both the US-VAE and surgery groups. We performed the analysis without it and found no difference in the original results (**Supplementary Material**). In addition to this limitation, our results are still of value once the mean time to recurrence ranging from 17.4 to 36 months is in accordance with the literature (21).

## Conclusion

In summary, there are no randomized data, nor are there ever likely to be, comparing US-VAE, margins < 1 cm, and margins > 1 cm for surgical treatment of benign PT. Based on these meta-analyses and other observational data, US-VAE appears to be as safe as surgery for the treatment of benign phyllodes tumors, with no difference in local recurrence, and should be an option in the era of personalized treatment. Prospective registration of long-term outcomes is, however, important to further determine the applicability of US-VAE in specific subgroups. These findings are not applicable to borderline or malignant PT, and surgery with at least 1 cm of margin width is still the treatment of choice.

## Data availability statement

The original contributions presented in the study are included in the article/**Supplementary Material**. Further inquiries can be directed to the corresponding author.

## Author contributions

MG: Writing – review & editing, Writing – original draft, Visualization, Validation, Supervision, Software, Resources, Project administration, Methodology, Investigation, Funding acquisition, Formal analysis, Data curation, Conceptualization. BAC: Writing – review & editing, Writing – original draft, Visualization, Validation, Supervision, Software, Resources, Project administration, Methodology, Investigation, Funding acquisition, Formal analysis, Data curation, Conceptualization. HC: Writing – review & editing, Writing – original draft. HS: Writing – review & editing. EP: Writing – review & editing. NS: Writing – review & editing. RM: Writing – review & editing. SM: Writing – review & editing. PD: Writing – review & editing. FC: Writing – review & editing. GG: Writing – review & editing. ADS: Writing – review & editing. WA: Writing – review & editing. JA: Writing – review & editing. CL: Writing – review & editing. ALSF: Writing – review & editing, Writing – original draft, Visualization, Validation, Supervision, Software, Resources, Project administration, Methodology, Investigation, Funding acquisition, Formal analysis, Data curation, Conceptualization.
