# Supplementary figures and images for: Vacuum-assisted excision: a safe minimally invasive option for benign phyllodes tumor diagnosis and treatment—a systematic review and meta-analysis
Source: Front Oncol. 2024 May 14;14:1394116. doi: 10.3389/fonc.2024.1394116 (PMC11130386; doi:10.3389/fonc.2024.1394116)

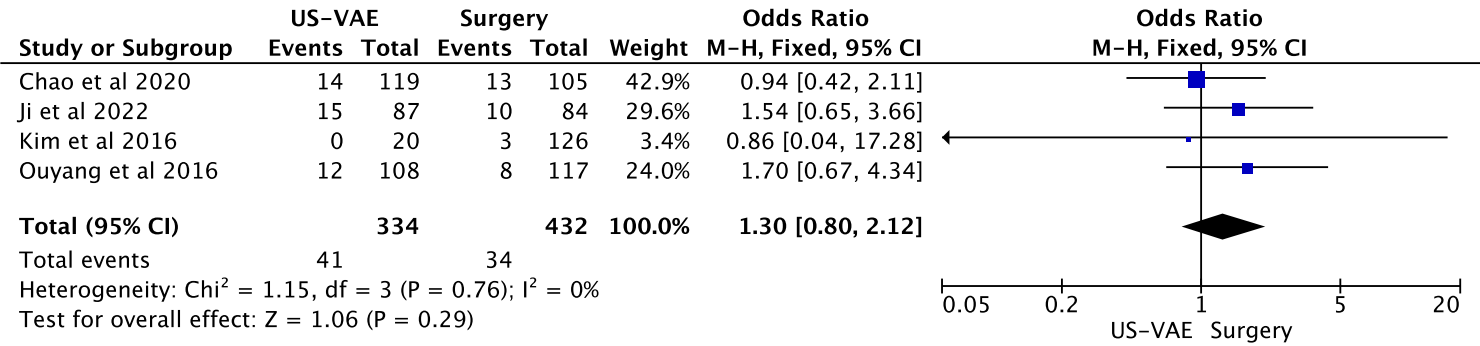

Supplement: Supplementary file 1 [file DataSheet_1.pdf]
