# Supplementary material for: Vacuum-assisted excision: a safe minimally invasive option for benign phyllodes tumor diagnosis and treatment—a systematic review and meta-analysis
Source: Front Oncol. 2024 May 14;14:1394116. doi: 10.3389/fonc.2024.1394116 (PMC11130386; doi:10.3389/fonc.2024.1394116)
Supplement: Supplementary file 2 [file Table_1.docx]

| **Section and Topic** | **Item #** | **Checklist item** | **Location where item is reported** |
| --- | --- | --- | --- |
| **TITLE** | | |  |
| Title | 1 | Vacuum Assisted Excision (VAE): a safe minimally invasive option for benign Phyllodes Tumor Diagnosis and Treatment: a systematic review and Meta-analysis | Page 01, detail “Title” |
| **ABSTRACT** | | |  |
| Abstract | 2 | This is a systematic review and meta-analysis comparing surgical excision and percutaneous ultrasound-guided vacuum assisted excision (US-VAE) for treatment of benign phyllodes tumor (PT) using local recurrence (LR) as the endpoint.  Objective: To determine the frequency of LR of benign PT after US-VAE compared to the frequency of LR after surgical excision. Method: Systematic review and meta-analysis (following the PRISMA standard) comparing LR in women older than 18 years old treated for benign PT by US-VAE compared with local surgical excision, with at least 12 months follow up. Studies were retrieved from PubMed, Scopus, Web of Science and Embase. The inclusion criteria was: manuscripts that described randomized clinical trials, comparative observational prospective and retrospective studies, clearly presenting the rate of local recurrence for both groups. The pooled effect measure used was the odds ratio (OR) of recurrence. Results: Five comparative prospective or retrospective observational studies published between 01/01/1992 and 01/10/2022, comparing surgical excision and percutaneous ultrasound-guided vacuum assisted excision (US-VAE) for local recurrence (LR) of benign phyllodes tumor (PT) met the selection criteria. Four were retrospective observational cohorts and one was a prospective observational cohort. In total 778 women were followed. Of these, 439 (56,4%) underwent local surgical excision and 339 (43,6%) patients had US-VAE. The median age of patients in the 5 studies ranged from 33,7 to 39 years old; the median size ranged from 1,5cm to 3,0cm and the median follow up ranged from 12 to 46,6 months. Needle gauge ranged from 7G to 11G. LR rates were not statically significant between US-VAE and surgical excision (41 of 339 versus 34 of 439; OR 1.3; p = 0.29). Conclusion: This meta-analysis suggests that using US-VAE for removal of benign PT does not increase local regional recurrence of benign PT and is a safe minimally invasive therapeutic option. | Page 01, detail “Abstract” |
| **INTRODUCTION** | | |  |
| Rationale | 3 | Many studies have shown that complete removal of benign PT using percutaneous excision is feasible. With this procedure PT are removed sample by sample in a piecemeal fashion using 7 to 11 G vacuum assisted needles under ultrasound guidance (US-VAE). It was postulated that a ´´wait and watch`` approach after vacuum excision (without evident clear margins) could be a safe alternative to local surgical excision with low LR. Indeed, complete percutaneous removal would safely exclude a malignant diagnosis and may result in acceptable local control for benign PT. | Page 01-02, detail “Introduction” |
| Objectives | 4 | To determine the frequency of LR of benign PT after US-VAE compared to the frequency of LR after surgical excision. | Page 01-02, detail “Introduction” |
| **METHODS** | | |  |
| Eligibility criteria | 5 | Manuscripts that described randomized clinical trials, comparative observational prospective and retrospective studies, clearly presenting the rate of local recurrence for both groups, were selected. Duplicate articles were removed, and conference summaries, editorials, comments, letters, and case reports were excluded. Only articles where complete US-VAE resection was defined as the complete removal of the lesion, verified by clinical examination (absence of palpable nodule) and ultrasound (absence of any lesion visualized in real-time examination) were selected. A clinically palpable lesion in the breast or its observation on ultrasound in previous tumor resection bed was considered a local recurrence. Lesions more than 2 cm away from the original tumor were not considered recurrences. Therefore, only studies that provided sufficiently detailed information were included. | Page 03, detail “Methods” – “Study selection” |
| Information sources | 6 | A broad search in PubMed, Scopus, Web of Science, and Embase databases using these keywords combined to Boolean AND or OR, for articles published between January 01/01/1992 and 01/10/2022 was done in 01/11/2022. There was no language restriction. | Page 03, detail “Methods” – “Search strategy” |
| Search strategy | 7 | The following keywords, terms and their combinations were used to formulate the search strategy: "Phyllodes tumor", phylloid tumor, vacuum-assisted excision, vacuum biopsy, and vacuum-assisted biopsy. The research was limited to human studies. There was no language restriction. | Page 03, detail “Methods” – “Search strategy” |
| Selection process | 8 | The studies identified in the initial search were imported in the Rayyan app for sorting and selecting articles 17. Two researchers were independently and blindly selected relevant articles using Rayyan. Initially, the studies were grouped, duplicate articles were removed, and conference summaries, editorials, comments, letters, and case reports were excluded. Discordance in the selected articles were resolved by consensus, and when this was not obtained a third researcher acted as arbiter. The complete papers of potentially relevant articles were retrieved and evaluated for eligibility. Duplicated papers were removed.  Only articles where complete US-VAE resection was defined as the complete removal of the lesion, verified by clinical examination (absence of palpable nodule) and ultrasound (absence of any lesion visualized in real-time examination) were selected. A clinically palpable lesion in the breast or its observation on ultrasound in previous tumor resection bed was considered a local recurrence. Lesions more than 2 cm away from the original tumor were not considered recurrences. Therefore, only studies that provided sufficiently detailed information were included. The two researchers, independently, decided on the inclusion or exclusion of studies, based on these predefined inclusion and exclusion criteria, again a third researcher acted as arbiter. Reasons for the exclusion of any article were documented. | Page 03, detail “Methods” –  “Study selection” |
| Data collection process | 9 | Data from eligible studies were extracted independently by the researchers using a standard form for data extraction developed for this review. The form contains the characteristics of the study (design), of the participants (sample size, age), interventions, outcomes assessed and the duration of follow-up (see supplemental materials). | Page 04, detail “Methods” –“ Data extraction” |
| Data items | 10a | Local recurrence was compared in women older than 18 years old treated for benign PT by US-VAE or by local surgical excision, with at least 12 months of follow-up. | Page 02-03, detail “Methods” –“Study design and selection criteria” |
|  | 10b | Age (years), time to recurrence (months), US-VAE tumor size (median / cm), surgery tumor size (median / cm). | Page 04, detail “Methods” –“Data extraction” |
| Study risk of bias assessment | 11 | The risk of bias in the studies was assessed through the specific forms from the Joanna Briggs cohort study Institute - JBI Critical Appraisal checklist, 2020 18. The analysis of the publication bias was carried out through the inspection of data asymmetry, according to dispersion and size of the effect in the Funnel Plot. | Page 04, detail “Methods” –“Assessment of methodological quality - Risk of bias” |
| Effect measures | 12 | According to the binary variable analyzed, local recurrence (LR) of benign PT and odds ratio (OR) were calculated as an effect estimation measure. To this purpose, the Mantel-Haenszel test was used. A fixed or random effect was applied according to the heterogeneity index found. | Page 04, detail “Methods” –“ *Statistical analysis****”*** |
| Synthesis methods | 13a | Manuscripts that described randomized clinical trials, comparative observational prospective and retrospective studies, clearly presenting the rate of local recurrence for both groups, were selected. | Page 03, detail “Methods” –“select studies” |
|  | 13b | The studies identified in the initial search were imported in the Rayyan app for sorting and selecting articles. Two researchers were independently and blindly selected relevant articles using Rayyan. Initially, the studies were grouped, duplicate articles were removed, and conference summaries, editorials, comments, letters, and case reports were excluded. Discordance in the selected articles were resolved by consensus, and when this was not obtained a third researcher acted as arbiter. The complete papers of potentially relevant articles were retrieved and evaluated for eligibility. Duplicated papers were removed. | Page 03, detail |
|  | 13c | Three studies (Chao et al. 2020, Vargas et al. 2006, and Ji et al. 2022) did not present the BI-RADS classification of the lesions. Ouyang et al. 2016 reported most patients had BI-RADS 4 lesions in the surgery group (58.1%) and Kim et al. 2016 reported (57.1%). In the US-VAE group, most lesions were classified as BIRADS 2-3 (58.3%) in Ouyang et al. 2016 and 50% in the study by Kim et al. 2016. Only one study presented the BI-RADS classification of the relapse (Kim et al. 2016). All recurrences of this study occurred in the surgery group and were classified as BI-RADS 4.  In the US-VAE group (n=339), local recurrence was observed in 41 (12,1%) cases and in the surgical group (n= 439), 34 (7,7%) cases. There was no significant difference in benign PT local recurrence regardless of the procedure performed (OR=1.30; P=0.29) (Fig 2). | Page 04-05, detail “Results*” and Table 01* |
|  | 13d | According to the binary variable analyzed, local recurrence (LR) of benign PT and odds ratio (OR) were calculated as an effect estimation measure. To this purpose, the Mantel-Haenszel test was used. A fixed or random effect was applied according to the heterogeneity index found. All analyses were performed on RevMan Web. | Page 04, detail “*Statistical analysis”* |
|  | 13e | For evaluation of heterogeneity, Higgins' heterogeneity classification was used. I2 < 40% represents inexpressive heterogeneity whereas values above 75% would indicate high heterogeneity. Following the recommendations, the statistical analysis was adjusted according to the level of heterogeneity, applying fixed effects model for inexpressive heterogeneity (low) and a random effects model for high heterogeneity. | Page 04, detail “*Statistical analysis”* |
|  | 13f | There is no sensitivity analysis. | - |
| Reporting bias assessment | 14 | The risk of bias in the studies was assessed through the specific forms from the Joanna Briggs cohort study Institute - JBI Critical Appraisal checklist, 2020 18. The analysis of the publication bias was carried out through the inspection of data asymmetry, according to dispersion and size of the effect in the Funnel Plot. | Page 04, detail “Methods” - “*Assessment of methodological quality* - *Risk of bias"* |
| Certainty assessment | 15 | According to the binary variable analyzed, local recurrence (LR) of benign PT and odds ratio (OR) were calculated as an effect estimation measure. To this purpose, the Mantel-Haenszel test was used. A fixed or random effect was applied according to the heterogeneity index found. All analyses were performed on RevMan Web. For evaluation of heterogeneity, Higgins' heterogeneity classification was used. I2 < 40% represents inexpressive heterogeneity whereas values above 75% would indicate high heterogeneity. Following the recommendations, the statistical analysis was adjusted according to the level of heterogeneity, applying fixed effects model for inexpressive heterogeneity (low) and a random effects model for high heterogeneity 18. | Page 04, detail “Methods” - “*Statistical analysis”* |
| **RESULTS** | | |  |
| Study selection | 16a | A total of 1254 references published between 01/01/1992 and 01/10/2022 were identified. Reviews, conference abstracts, editorials, commentaries, letters, and case reports were also excluded (n= 697). Titles and abstracts were examined to remove articles that were not related to the topic (n=546). The full texts of potentially relevant articles (n=11) were retrieved, and the eligibility criteria were evaluated. Six articles were excluded, 5 did not present the comparator group (surgery) and/or presented incomplete data on recurrence rates following surgery. One was published in a Chinese periodic that was not available. At last, all 5 articles selected were observational cohorts, 4 retrospective and one prospective (Fig. 1). Four papers were from Asia, three from China and one from Korea, and one from USA (table 1). | Page 04-05, detail – “Results” and figure 01 |
|  | 16b | Six articles were excluded, 5 did not present the comparator group (surgery) and/or presented incomplete data on recurrence rates following surgery. One was published in a Chinese periodic that was not available | Page 04-05, detail – “Results” and figure 01 |
| Study characteristics | 17 | All the included studies and their characteristics are in the Table 01. | Page 06, detail “Results” - figure 01 |
| Risk of bias in studies | 18 | All the assessments of risk of bias for each included study are in Figure 2 - Forest Plot. | Page 07, detail “Results” - figure 02 |
| Results of individual studies | 19 | It can be found in the Table1, Figure 02, and in the text. | Pages 04-07, detail “Results” - figure 02 |
| Results of syntheses | 20a | It can be found in the table 01 and table 02, | Page 06-07, detail “Results” - table 01 and 02, |
|  | 20b | It can be found in the Figure 02 | Page 07, detail “Results” - figure 02 |
|  | 20c | It can be found in the figure 02. | Page 07, detail “Results” - figure 02 |
|  | 20d | It can be found in the figure 02. | Page 07, detail “Results”- Figure 02 |
| Reporting biases | 21 | There was no significant publication bias, according to individual analysis of the studies (table 2) and by visual inspection of the Funnel Plot (Fig 3), which shows no asymmetry of the studies in their positions in the funnel. One of the studies was not plotted, because no case of recurrence occurred, in both conditions (US-VAE and surgery). | Page 07, detail “Quality analysis and risk of bias”, Table 02 and figure 03 |
| Certainty of evidence | 22 | It can be found in the table 02. | Page 07, detail “Results” - table 02 |
| **DISCUSSION** | | |  |
| Discussion | 23a | There are no randomized data, nor are there ever likely to be, comparing US-VAE, margins<1cm and margins >1cm for surgical treatment of benign PT. Our meta-analysis supports US VAE as an alternative approach to surgery for benign PT as there is no significant difference in LR after resection by either surgical excision or US-VAE. The current diagnosis and management of PT are based on observational data 1-9. Pathologists find it challenging to exclude malignant phyllodes on core needle biopsy (CNB)/vacuum assisted biopsy (VAB) sampling and tend to classify them as fibroepithelial lesions. Current practice consists of excisional surgery with clear margins, but not 2cm wide since most of these cases are fibroadenoma or benign PT9. There is emerging data supporting the so called ´´wait and watch`` approach that consists of follow up and observation after a surgical excision with positive margins or an US-VAE 10-15,19, and our meta-analysis corroborates this. | Page 08, detail “Discussion” |
|  | 23b | Still, the studies are all non-randomized cohorts and therefore there is the potential for selection bias in the cases having US-VAE versus those having surgery. | Page 11, detail “Discussion” |
|  | 23c | There are some limitations to our meta-analysis. Only two studies reported median tumour size in the two groups, 1,7cm for US-VAE/3,0cm for surgery (Ouyang et al. 2016) and 1,6cm for US-VAE/3,0cm for surgery (Kim et al. 2016). Although the median tumor size was smaller in the US-VAE groups than surgery, there were no associations between tumor size and local recurrence. Still, the studies are all non-randomized cohorts and therefore there is the potential for selection bias in the cases having US-VAE versus those having surgery.  Furthermore needle sizes between studies were different. One study used 7-gauge needles (G), two used 11G or 8G, one used 8G and in one it was not specified. In Vargas et al. 2006, the 8G needle was used in 169 patients and the 11G needle was used in 41 patients. One study (Ouyang et al. 2016) used only 8G needle. It was not possible to determine the relation between the gauge needle and LR. Probably, the gauge of the needle used doesn´t matter if complete excision is achieved.  Finally, the recurrence intervals between the US-VAE and surgery groups described in Table 1 are in general relatively short and not well described. The study by Chao et al. 2020 presents only the mean overall recurrence interval. It does not differentiate it by groups. Ji et al.2022 presented only the mean overall recurrence interval (36 months) and did not differentiate it by groups. In the meta-analysis of Yiwen Lu at all, the median time to recurrence was longer than 24 months in nine studies and shorter than 24 months in eight studies, one of the studies presented median local recurrence time of 6 months19. In our meta-analysis the median follow up time from the studies ranged from 12 to 48,6 months and mean time to recurrence ranged from 17,4 to 36 months. In one of the studies the follow up time was just 12 months but showed no recurrence at all in both US-VAE and surgery group. We did the analysis without it and showed no difference in the original results (supplemental material). | Page 11, detail “Discussion” |
|  | 23d | Based on this meta-analyses and other observational data, US-VAE appears to be as safe as surgery for treatment of benign phyllodes tumor, with no difference in local recurrence, and should be an option in the era of personalized treatment. Prospective registration of long-term outcomes is, however, important to further determine the applicability of US-VAE in specific subgroups. | Page 11-12, detail Conclusion |
| **OTHER INFORMATION** | | |  |
| Registration and protocol | 24a | Protocol CRD 42022309782.. | Page 01-02, detail “Methods” - *“register”* |
|  | 24b | International Prospective Register of Systematic Review (PROSPERO) | Page 01-02, detail “Methods”- *“register”* |
|  | 24c | There was a change in the title of the Systematic Review. Now it is Vacuum Assisted Excision (VAE): a safe minimally invasive option for benign Phyllodes Tumor Diagnosis and Treatment - a systematic review and meta-analysis. | Page 01-02, detail “Methods”- *“register” and* Page 01, detail “Title”. |
| Support | 25 | Financial support for the review: None | Page 12, detail “Financial support for the review: None” |
| Competing interests | 26 | Declarations of interest referring to all authors: “none” | Page 12, detail “Declarations of interest referring to all authors: none” |
| Availability of data, code and other materials | 27 | Publicly available can be found on International Prospective Register of Systematic Review (PROSPERO) - Protocol CRD 42022309782: data extracted from included studies and the search strategy. The selected studies are available in the reference list. | Page 01-02, detail “Methods”- *“Register” and* Page 12, detail “References” |

*From:*  Page MJ, McKenzie JE, Bossuyt PM, Boutron I, Hoffmann TC, Mulrow CD, et al. The PRISMA 2020 statement: an updated guideline for reporting systematic reviews. BMJ 2021;372:n71. doi: 10.1136/bmj.n71

For more information, visit: <http://www.prisma-statement.org/>
