# Supplementary material for: Vacuum-assisted excision: a safe minimally invasive option for benign phyllodes tumor diagnosis and treatment—a systematic review and meta-analysis
Source: Front Oncol. 2024 May 14;14:1394116. doi: 10.3389/fonc.2024.1394116 (PMC11130386; doi:10.3389/fonc.2024.1394116)
Supplement: Supplementary file 3 [file Table_2.docx]

Appendix B - Data Extraction Form:

ID – author, year of publication:

**A) CHARACTERISTICS OF THE STUDIES**

1. Design (type of study):

2. Study Period:

3. Country of Origin:

4. Institution of origin of the first author:

5. Publication Journal:

6. Statistical Analysis:

**B) PARTICIPANTS**

1. Sample size (n):

2. Age (average or median):

3. Tumor Size (cm):

4. BI-RADS classification of breast lesions:

5. Inclusion Criteria:

6. Exclusion Criteria:

**C) INTERVENTION**

1. Type of intervention:

2. Tracking time:

3. Evaluation moments:

**ASSESSED OUTCOMES AND OUTCOMES**

1. Primary outcome(s):

2. Secondary outcome(s):
